# Developing a design-based concept to improve hand hygiene in the neonatal intensive care unit

**DOI:** 10.1038/s41390-023-02482-9

**Published:** 2023-01-24

**Authors:** Sophie J. Jansen, Britt J. Müller, Sophie J. E. Cramer, Arjan B. te Pas, Enrico Lopriore, Vincent Bekker

**Affiliations:** 1 Division of Neonatology, Department of Pediatrics, Leiden University Medical Center (LUMC), Leiden, The Netherlands; 2 Department of Industrial Design Engineering, Delft University of Technology, Delft, The Netherlands

## Abstract

**Background:**

Hand hygiene (HH) is the most critical measure in the prevention of nosocomial infections in the neonatal intensive care unit (NICU). Improving and sustaining adequate HH compliance rates, however, remains a significant challenge. Using a behavioral change framework and nudge theory, we developed a design-based concept aimed at facilitating and stimulating HH behavior.

**Methods:**

Concept development was initiated by selecting a theoretical framework after which contextual field studies aimed at discovering causes for poor compliance were conducted. Potential solutions were brainstormed upon during focus group sessions. Low-fidelity prototypes were tested regarding feasibility, usability, and acceptability. A final concept was crafted drawing from findings from each design phase.

**Results:**

Complying with recommended HH guidelines is unrealistic and infeasible due to frequent competing (clinical) priorities requiring HH. The concept “Island-based nursing,” where a patient room is divided into two geographical areas, namely, the island and general zone, was created. HH must be performed upon entering and exiting the island zone, and after exposure to any surface within the general zone. Reminding of HH is prompted by illuminated demarcation of the island zone, serving as the concept’s nudge.

**Conclusions:**

Island zone demarcation facilitates and economizes HH indications in an innovative and intuitive manner.

**Impact:**

- Although hand hygiene (HH) is the single most important element in the prevention of nosocomial infections in neonates, improving and sustaining adequate HH compliance rates remains a significant challenge.
- Complying with recommended HH guidelines was found to be unrealistic and infeasible due to the significant amount of time required for HH in a setting with a high workload and many competing (clinical) priorities.
- The concept of “Island-based nursing,” under which the primary HH indication is upon entering and exiting the island zone, facilitates and economizes HH indications in an innovative and user-friendly manner.

## Introduction

Nosocomial infections (NI) continue to be a major health issue in neonatal intensive care units (NICU) worldwide, with the hands of nurses, physicians and allied-health personnel being the most common vehicle for the transmission of pathogenic microorganisms. Hand hygiene (HH) has consequently been singled out as one of the most critical measures in reducing the incidence of NI.^1–3^ However, despite significant advances in infection control and hospital epidemiology, compliance remains suboptimal and difficult to sustain, often lingering below 50%.^2,4,5^ Given that NIs are a major source of neonatal morbidity and mortality, as well as long-term neurodevelopmental and growth impairment, prolonged hospital stay and increased healthcare expenditures, heightened efforts to enable continued improvements in reducing the burden of NI are urgently needed.

Over the past decade, a multitude of (non-)technical interventions have been developed and implemented as a means to improve adherence to HH in critical-care settings.^6,7^ Conventional strategies such as improving the accessibility and availability of alcohol-based hand rub dispensers, educational campaigns and performance feedback have, however, proven to be insufficient.^8,9^ An approach that has steadily gained popularity as a behavioral change tool is the so-called “nudge,” or subtle intervention that alters the decision environment and is founded upon the notion that biases and flaws are present in human rational decision-making.^10,11^ Despite the relative paucity of evidence on the effectiveness of nudging to improve HH compliance, numerous studies have successfully demonstrated the effectiveness of nudging within the healthcare realm. In a systematic review conducted by Nagtegaal et al., the majority (77%) of studies evaluating the effect of HH nudging were successful, making HH the most effective behavioral outcome of the review.^12^ However, there was little variation with respect to the type of nudge tested, with nearly all nudges consisting of simple alterations in the location of alcohol-based hand rub dispensers.^12^ Given that optimal adherence to HH is dependent on ward-specific microenvironments, processes and task requirements, a system which departs from the traditional and attempts to mitigate barriers by considering human cognitive and physical strengths may therefore be beneficial.

An assessment of nosocomial bloodstream infection rates between 2012 and 2020 at our NICU revealed a mean 9-year incidence rate of 9.25 per 1000 patient-days among preterm infants (<32 weeks’ gestation).^13^ The supplementary finding that 66% of these infections were caused by coagulase-negative staphylococci (CoNS), the prevention of which is strongly enhanced by appropriate HH, stimulated the need for further assessment of (baseline) HH compliance rates. Based on a HH compliance measurement, an overall HH compliance rate of 49% was found. Opportunities before patient contact, after patient contact and after contact with patient surroundings were missed most frequently. These results subsequently formed the point of departure for further process improvement.

Rather than concentrating on conventional approaches or further modifying and refining existing infection prevention guidelines, we sought to develop and implement a novel design-based concept aimed at facilitating and stimulating HH behavior by identifying barriers and optimizing the environment and natural work processes. The purpose of the present study is to therefore describe the design process of the intervention, the rationale for the included elements and its practical application.

## Methods

### Setting

This design-research project was conducted at The Leiden University Medical Center (LUMC), a university-affiliated teaching hospital providing tertiary medical care for residents of Leiden, The Netherlands and its surrounding area. The hospital contains a 25-bed level-III NICU divided among two units, with the first unit consisting of 9 single-patient rooms and 2 twin rooms, and the second containing 8 single-patient rooms and 2 twin rooms. Approximately 120 healthcare professionals, consisting of nurses, neonatologists, pediatric-residents, and allied-health staff (i.e., researchers and housekeeping staff) work in the NICU. Participation in this project was limited to the NICU healthcare workers. A research protocol was developed and approved by the institutional review board of the LUMC as part of a quality assurance program prior to the initiation of the study (N20.087).

### Design methods

Five methods were used to gather information on contextual factors, identify barriers to performing HH, design and validate interim design concepts, and establish a final concept aimed at process optimization:

1. Selection of a theoretical framework used as the point of departure for the design process.
2. Contextual field studies consisting of empirical observations of the daily workflow of NICU healthcare workers in relation to features that positively or negatively influence HH performance and compliance.
3. Focus groups with NICU healthcare workers to elicit feedback on the suitability of proposed interim design concepts considering barriers and contextual factors identified in the previous method.
4. Iterative prototype testing in which five preliminarily concepts were tested and information regarding their feasibility, applicability and usability was gathered.
5. Development of the final concept based on the findings from the previous method.

### Theoretical framework

Numerous studies that have proposed theory-driven interventions to improve HH compliance have used the Theory of Planned Behavior (TPB) as a theoretical framework.^14,15^ The TPB provides a systematic overview of constructs that are believed to predict intention to perform a certain behavior. These constructs include (i) attitude towards the behavior, (ii) social pressure individuals perceive themselves to be under to perform the behavior, and (iii) extent to which individuals believe they can adequately perform the behavior.^16^

**Fig. 1:**
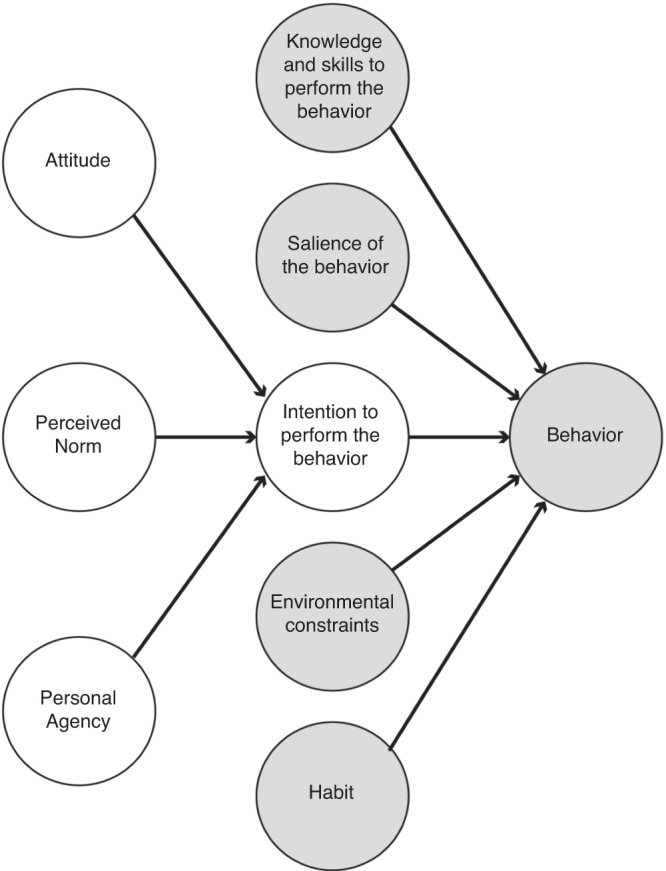
The Integrated Behavior Model. The model features the original as well as  four additional constructs that directly affect behavior.^17^

To extend the TPB to infer internal and external factors that influence HH performance, we used the Integrated Behavior Model (IBM), an evolution of the TPB that integrates four additional constructs that directly affect behavior, namely (i) knowledge and skills to perform the behavior, (ii) salience of the behavior, (iii) environmental constraints, and (iv) force of habit.^16,17^ The model provides an inclusive and extensive theoretical framework that captures all aspects deemed relevant to facilitate and induce HH behavior (Fig. 1). This framework was hence used to pinpoint perceived cognitive and physical factors that affect HH compliance of NICU healthcare workers as previously established in the current literature.

### Contextual field studies 1: evaluation of trends and causes for non-compliance

To discover trends and root causes for poor compliance and understand decision-making rationale, contextual field studies, consisting of observing and shadowing a variety of NICU healthcare workers, were conducted. Shadowing sessions were performed by one member of the research team without former experience in the hospital setting (BM), allowing for objective observations regarding HH compliance. Individuals subject to observation were informed about the shadowing sessions but not the specific aim or the nature of the observations. Insights gathered during the field studies were used to assess literature-based factors that affect HH compliance within their contextual relevance. The IBM was subsequently supplemented where necessary and factors deemed irrelevant were removed. All observations and findings were documented in qualitative descriptions.

### Focus groups

Four focus groups with 8, 5, 9, and 6 participants, respectively, were held to gain rich insights into healthcare workers’ perceptions of HH compliance, validate observed barriers in performing HH in the current workflow, and brainstorm solutions as to how these barriers could be addressed. Convenience samples of NICU nurses and physicians available for the educational gatherings starting prior to their work shift were included. Sessions were supervised by two researchers (S.J.J., B.J.M.) guiding the unstructured, interactive sessions, with both moderators having formal training in qualitative research methods (including designing and guiding focus groups) and one moderator (B.J.M.) having expertise in user-experience design and design-thinking. Further details regarding the content and procedural aspects of the focus group sessions can be found in Supplementary Material 1.

### Prototype testing

Using results from the field studies and focus groups, five interim design solutions were developed and subsequently tested regarding their feasibility, usability and acceptability within their intended context (i.e., during patient care-related activities in a single-patient room or twin room) by means of low-fidelity prototypes. The prototypes were individually tested during five different simulation sessions (i.e., not during actual patient care), with qualitative data gathered from a total of 56 participants (*n* = 7, 15, 10, 11, 13, respectively) consisting of nurses and physicians working during the corresponding day shift. In-context, semi-structured interviews and observations based on a pre-determined (clinical) scenario in which participants were asked to perform a task in line with their function (i.e., feeding, changing diapers, administering medication, performing a physical examination) were undertaken to understand to what extent the proposed concepts formed workable solutions. Topic lists were developed beforehand and amended throughout this study phase to include issues that emerged as important during initial testing sessions. Participants were likewise encouraged to freely express their thoughts about the prototypes. Interviews were held until data saturation was reached. All remarks and insights were documented and used for development of the final concept.

### Contextual field studies 2: assessment of the practicability of current HH guidelines

To quantify the extent to which the current HH guidelines are feasible and practicable in the current workflow, three supplementary shadowing sessions were held in which NICU healthcare workers were observed during performance of standard daily tasks. Sessions were held during three separate day shifts in which nurses and physicians were observed. Each shift was divided into half-hour intervals for which the absolute number of required HH indications was recorded, regardless of whether the indication was effectuated upon or not. Only time intervals where healthcare workers were active in patient care-related tasks were observed. All participants were informed about the nature of the observations given that the aim of the sessions was not to measure HH compliance but merely quantify the hypothetically required moments in which HH would need to be performed.

## Results

The abovementioned methods formed the basis of an overall iterative design process. Below we provide a description of each phase of the design process and its accompanying findings.

### Contextual field studies 1: trends and causes for HH non-compliance

A total of 40 h, spread out over 5 day shifts, were spent directly observing nursing and physician staff members. Insights obtained during the field studies primarily involved defining which HH opportunities were most appropriate to be set as intervention targets. Thematic analysis of observational notes revealed that NICU healthcare workers had the most difficulty complying with HH recommendations when performing tasks in and around the incubator during patient care (i.e., adjusting ventilator settings, opening the bedside drawer to grab an item or charting patient data on the bedside computer). These (non)clinical interruptions require HH, thereby prolonging the time needed to complete regular clinical tasks. These findings are in alignment with results from the previously performed baseline HH compliance assessment, illustrating that HH moments 1 (before patient contact), 3 (after patient contact) and 5 (after contact with patient surroundings) are the most frequently missed opportunities among our healthcare staff (Table 1).

**Table 1:** Hand hygiene non-compliance rates among NICU healthcare workers.

Moment	Non-adhered/total opportunities	Percentage
Total	127/249	51%
1. Before patient contact	40/95	42.1%
2. Before clean/aseptic procedures	3/10	30%
3. After body fluid exposure/risk	6/6	100%
4. After patient contact	54/97	55.7%
5. After patient surroundings	24/41	58.5%

*NICU* neonatal intensive care unit.

### Preliminary focus: optimizing salience of the behavior

Based on the results obtained from the field studies, salience of the behavior was subsequently designated as a key factor that prohibits appropriate HH in our NICU. According to this model component, a behavior must be deemed sufficiently important, and actively thought of, for it to be consistently and properly carried out.^18^ An intervention which provides visual feedback on the one hand, and initiates active thinking about HH before and after immediate patient contact on the other, could be efficiently addressed by means of design.

### Focus groups

The sessions revealed insights into how potential barriers could be addressed and which adjustments to the proposed concepts should be made. For example, it was suggested that the floor projection surrounding the incubator needed to be of sufficient size to be visible when performing care tasks inside the incubator (Supplementary Material 2B). Similarly, it was noted that it may feel counterintuitive to introduce a projected (floor) light surrounding the incubator while the NICU environment strives to be as minimally sensitizing for the infant as possible. Focus groups also yielded important insights regarding the timing of the nudge, with several healthcare workers proposing the incorporation of an adjustable timer during which the nudge could be silenced when performing acute tasks composed of many consecutive activities. Although auditory and olfactory sense-scapes were also proposed, these were perceived negatively by the participants and therefore abandoned as potential intervention components. As such, a nudge which does not result in unwarranted sensory stress and contains accurate trigger timing was therefore considered imperative.

**Fig. 2:**
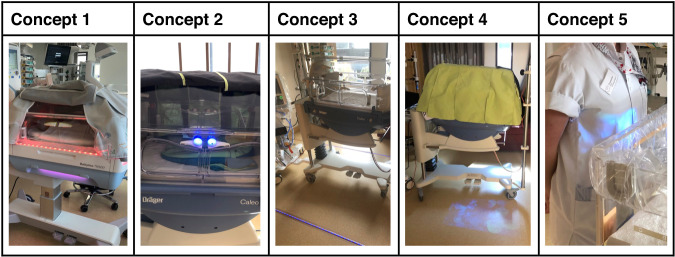
Low-fidelity prototypes of the five preliminary concepts. Each individual concept features a dynamic light source yet differs regarding the location of the light source, type of feedback given and timing of the intervention.

Analysis of focus group data resulted in the development of five interim design concepts (Table 2 and Fig. 2). Each concept featured a dynamic light source (i.e., a light changing in hue or intensity) but differed with regard to the location of the light source, type of feedback given and optimal timing of the intervention.

**Table 2:** Five interim design concepts, each featuring a dynamic light differing in location, type of feedback, and timing of the intervention.

	Concept 1	Concept 2	Concept 3	Concept 4	Concept 5
Location					
Function	Light strip surrounding the incubator	Opening mechanism with built-in lights	Light strip surrounding the incubator	Dynamic projection surrounding the incubator	Dynamic projection visible on HCW himself/herself
Type of feedback	Changing hue (red > green)	Turning on/off (blue)	Turning on/off (blue)	Moving elements and turning on/off	Moving elements
Timing of the intervention	After HH moment	When hands are in close proximity; before HH moment	When entering/leaving the patient room	When entering/leaving the patient room	When in close proximity of the incubator

*HCW* healthcare worker, *HH* hand hygiene.

### Prototype testing

The feasibility, usability, and acceptability of the five interim design concepts were tested using low-fidelity prototypes (Fig. 2), with each individual testing session yielding numerous important design-related insights. First, a nudge which is simultaneously highly visible and not distracting or physically hindering during work-related activities was preferred. Similarly, colors on the lower end of the color spectrum (i.e., green and/or blue) were deemed more appeasing and less retaliative than the color red. Considerable agreement existed regarding the intensity of the light, which was requested to be of lower intensity to prevent distraction for all individuals present in the patient-room (healthcare workers, patients and parents). Moreover, both the timing and sustainability of the alerting aspect of the nudge were important, indicating that potential feedback should only be given prior to the required HH moments and contain sufficient visual stimulation to prevent habituation.

Qualitative data likewise revealed important feasibility-related insights. Healthcare workers indicated that a description of the nudge’s purpose (i.e., cognitive link between the nudge and HH behavior) prior to full implementation is necessary for proper execution. In addition, there was universal consensus that complying with all five HH moments was unrealistic and infeasible due to the presence of competing priorities, particularly in situations where acute care must be delivered rapidly. It appeared that healthcare workers often found it difficult to find a balance between performing HH and delivering immediate critical care.

Overall, prototype testing sessions demonstrated that although salience of the behavior could be increased through the implementation of a nudge, the implementation alone would be insufficient for improving HH compliance due to the unworkable nature of current HH recommendations which do not take into account the presence of a high workload and (non)clinical interruptions in daily practice, resulting in frequent abandonment of HH indications perceived as impracticable under time sensitive conditions.

### Contextual field studies 2: impracticability of current HH guidelines

During the supplementary contextual field studies, a total of 15 intervals were recorded (Table 3). Shadowing revealed that healthcare workers were hypothetically required to disinfect their hands a maximum average of 25 times per hour. Considering an application time of 30 s per HH opportunity, hand disinfection would have consumed an average of 12.5 min or 41.6% of the total time spent with the patient, highlighting the impracticality of full compliance with the WHO’s HH recommendations in acute care settings and providing a potential explanation for our previously found low HH compliance rate.

**Table 3:** Average number of observed required HH moments according to the WHO’s five moments of HH.^28^

Session 1		Session 2		Session 3	
Interval	Frequency	Interval	Frequency	Interval	Frequency
7:30–7:59	12	7:30–7:59	6	7:30–7:59	21
8:00–8:29	28	8:00–8:29	17	8:00–8:29	16
8:30–8:59	13	8:30–8:59	11	8:30–8:59	9
9:00–9:29	3	9:00–9:29	13	9:00–9:29	8
9:30–9:59	15	9:30–9:59	15	9:30–9:59	25
10:00–10:29	9	10:00–10:29	0	10:00–10:29	19
10:30–10:59	27	10:30–10:59	11	10:30–10:59	19
11:00–11:29	7	11:00–11:29	13	11:00–11:29	10
11:30–11:59	5	11:30–11:59	8	11:30–11:59	3
12:00–12:29	16	12:00–12:29	13	12:00–12:29	34
12:30–12:59	15	12:30–12:59	4	12:30–12:59	7
13:00–13:29	21	13:00–13:29	0	13:00–13:29	5
13:30–13:59	0	13:30–13:59	15	13:30–13:59	21
14:00–14:29	29	14:00–14:29	7	14:00–14:29	20
14:30–14:59	14	14:30–14:59	0	14:30–14:59	14
Total	214	Total	133	Total	231
Average per half-hour	14.27	Average per half-hour	8.86	Average per half-hour	15.4
Average per hour	26.75	Average per hour	17.73	Average per hour	30.8

*HH* hand hygiene.

### Shift in focus

Based on the above-mentioned findings, it was inferred that the largest barriers to HH compliance in the NICU consisted not only of salience of the behavior, but most notably process barriers in the form of competing priorities and (non)clinical interruptions, which consequently affect the personal agency of NICU healthcare workers. To optimize HH compliance, it was therefore concluded that the mere implementation of a nudge as a means of triggering and providing direct visual feedback on HH behavior turned out to be insufficient. Therefore, a different type of intervention is required that not only stimulates desired HH behavior elicited through the implementation of subtle environmental cues but also removes barriers to personal agency and simplifies the application of HH recommendations in the NICU context. This motivated us to develop a user-centered final concept consisting of the repurposing of HH recommendations in combination with a nudge.

### The final concept of Island-based nursing and its practical application

**Fig. 3:**
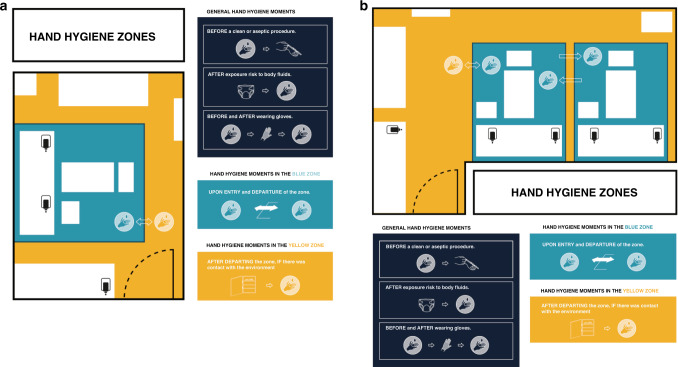
Final design concept of “Island-based nursing”. **a**, **b** Visualize the concept implemented in a single-patient room twin-room, respectively. Patient zone defined as the patient and his/her immediate surroundings colonized by a patient flora and healthcare zone containing all other surfaces within a single-patient or twin-room.

Our study identified frequent opportunities for HH and the infeasibility of standard guidelines, suggesting the need for a strategy which prioritizes the most essential HH moments and simplifies current protocols. With the aim of facilitating and economizing indications for HH in an innovative manner, we therefore chose to directly translate the evidence gathered above into a functional description and visualization of HH indications. The concept of “island-based nursing” was introduced in the form of a geographical visualization of principal indications for HH. Figure 3a, b illustrate the concept as implemented in a single-patient and twin room, respectively. In accordance with the concept, the patient room is divided into two geographical areas, namely, the blue “island zone” and the yellow “general zone.” The island zone contains the patient and his/her immediate surroundings, including all inanimate surfaces that are frequently touched by NICU healthcare workers or are in direct physical contact with the patient such as the incubator, bedside table, intravenous tubing, trash can, charting computer. Resident flora could potentially disseminate throughout the island zone, signifying that the zone has implications beyond HH to also include the decontamination of objects and environmental surfaces. The general zone contains all physical surfaces outside the island zone (i.e., the remaining room environment) consisting of the sink, countertop, cupboards with medical equipment, folding table and sofa-bed. The general zone is, conceptually, colonized with bacteria, including multidrug-resistant bacteria that may be potentially harmful to other patients as well and thereby should not be allowed to spread from patient to patient.

**Fig. 4:**
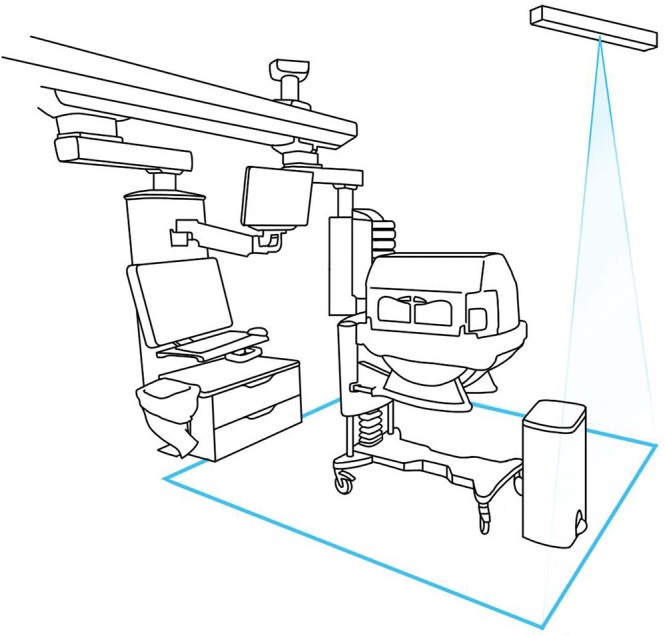
Nudging component of the “Island-based nursing” concept. Illuminated line, created by the laser projector suspended from the ceiling, demarcates the boundaries of the island zone.

Based on this concept, two principal HH indications can be inferred. The first indication is anchored upon entering (i.e., before touching the patient or any surface within the island zone) and exiting the island zone (i.e., after touching the patient or any surface of within the island zone and entering the general zone). This indication can be best visualized by crossing the illuminated line demarcating the island zone (Fig. 4). This illuminated demarcation, created by a laser projector suspended from the ceiling, serves as the concept’s nudge, reminding individuals that HH is required when crossing these boundaries. To prevent desensitization over time, the nudging mode (light intensity and color) can be adapted when desired. By performing HH upon entering the island zone, cross-transmission of microorganisms and potential exogenous infection is prevented while upon departing the zone contamination of an individual’s hands with patient flora is reduced, thus minimizing the risk of dissemination to the environment. When moving from one patient room to another without touching any surface outside the island zone, a single HH action suffices. This also applies when moving between two island zones in the same room: a single HH action between the two zones is sufficient.

The second indication for HH occurs after exposure to any surface within the general zone. In case of no hand exposure with objects within the general zone, no HH is indicated, as transmission risk of microorganisms that are foreign and potentially harmful to the patient is virtually absent. Alongside the two primary HH indications, three routines yet critical HH moments must always be performed regardless of the zone one is in (Fig. 4). Moreover, to assure successful execution of the concept, ergonomic localization of alcohol-based hand rub dispensers at the entrance of the patient room as well as on both sides of the incubator is essential.

## Discussion

Increasing evidence has indicated that interventions based on explicit theoretical foundations are more successful than those lacking a theoretical base, with several studies suggesting that failure to deliver high quality of care is often due to faulty design, whether device, process, or human factor related.^19–21^ The concept of HH has rarely been assessed from these perspectives until now, and relatively few studies have focused in detail on the practical issues associated with existing HH guidelines. Our island-based nursing concept was conceived as a result of the need to facilitate and economize indications for HH. By highlighting unique barriers to compliance and defining and allocating items to different geographic zones within a patient-room, in combination with instilling an environmental cue to trigger the desired behavior, we were able to reduce the need for excessive HH indications, thereby bridging the gap between existing HH guidelines and the need for a context-specific, user-friendly, and feasible solution.

Despite the increased number of publications on the effectiveness of HH interventions based on the WHO guidelines over the past two decades, the latter are subject to several limitations which have not been taken into account in the design and implementation of HH interventions. First, the guidelines do not consider the perspectives of healthcare workers, despite evidence that incorporation of their values and preferences are critical elements to achieve optimal HH compliance.^22^ Second, there are challenges associated with auditing all Five Moments due to poor visibility of activities within the patient zone (i.e., when bedside curtains obscure clinical activities), exclusion of visitors during auditing sessions and inability to capture certain, not directly visible, HH opportunities (i.e., after handling bodily fluids in utility rooms).^23^ Lastly and perhaps most importantly, HH is often compromised by a high workload and clinical competing priorities, making it difficult to determine when and how HH is to be performed. Evidently, modifying guidelines to meet contemporary and context-specific needs as well as integrating the perspective of the end-user, are therefore needed.

Elaboration of our island-based nursing concept with a nudge shows that interventions incorporating environmental modifications is a potentially promising avenue to guide HH behavior. Not only does zone demarcation offer clarification on which elements and items belong to a particular zone, but its primary function makes it act as a reminder for HH during point-of-care activities. Apart from the WHO’s inaugural study in which the Five Moments for HH are described within a time-space framework, little other evidence exists regarding the development and effectiveness of patient zoning.^24^ A study conducted by Yin et al. demonstrated significant improvement in HH compliance after the introduction of a low-tech solution of taping a patient zone demarcation in a pediatric intensive care unit.^25^ Other studies have incorporated the concept of patient zoning in more sophisticated interventions such as automated HH monitoring systems which generate reminding signals or computerized voice prompts based on predefined clinical zones.^26,27^ Evidently, simple yet sensitizing environmental features may be useful (and perhaps necessary) additions to increase salience of HH behavior.

Our concept largely relies on the premise that the concept was already implicitly being carried out, as evidenced by the most frequently missed HH moments (i.e., activities in and around the incubator during patient care). Our concept is thus no more than a mere explication and clarification of an existing natural workflow, supplemented by a nudge as a way of further enhancing the decision-making ability of the end-user. The heuristics of the concept made intuitive sense to all users, who were able to appreciate the function of the different zones and its elements as conceived by the research team. We presume that the concept was further propagated by terms such as “entering” and “exiting” the island zone, which promotes the idea that the zones are defined areas which must be entered and exited with a conscious mind regarding pathogenic transmission. However, our concept does not promote viewing all areas outside the island zone as a single, homogenous area, as this would unjustifiably oversimplify the complexity of the general zone. Maintaining proper awareness of the contamination risk of high traffic areas and elements operating as fomites, including their timely disinfection, is therefore essential.

### Strengths and limitations

Our design process should be interpreted in the context of several limitations. First, the design phases were limited to relatively small groups of participants from a single ward, restricting the generalizability of our concept. On the other hand, our concept consists of a simple, low-tech solution based on human factor science which may be applied to any clinical setting. Our concept can likewise be applied to multi-bed wards where infection control barriers may be less visible as compared to single-room wards. Ensuring continuous availability of HH supplies, regular training of standard infection prevention practices, organizational support and systematic feedback from management nonetheless form the basis for any additional, more intricate intervention and must therefore be considered prior to implementation in any setting. Our concept can nonetheless be taken as a conceptual catalyst to update HH practices in other clinical settings. Second, data collection and testing sessions had to be fitted into busy shifts, forcing us to keep the sessions short and limiting the number and variety of available test-users. Active participation of healthcare workers in the study nevertheless yielded unique insights into workable solutions tailored towards the natural workflow of NICU healthcare workers. Moreover, the sharing of a shared vision with active involvement throughout the entire design process allowed for the cultivation of a strong sense of ownership, acceptance, and commitment. Third, our concept was not designed to measure the quality of hand rubbing, despite this being another essential factor in preventing the cross-transmission of microorganisms. Development of interventions targeted at both the quantity and quality of HH performance are therefore recommended. Despite these limitations, our qualitative analyses and methodology allowed for an in-depth exploration of barriers and determinants of HH and the derivation of rich, context-specific insights necessary for the development of a targeted intervention. Likewise, considering that many care activities do not follow standard operating procedures, our concept allows for a simplification of HH auditing by limiting inter-observer variation and including all those present in the clinical environment (including visitors such as parents) in the auditing process.

## Conclusion

Stipulating behavior-, environmental-, and process-related determinants of HH practice and using the collective input from end-users allowed us to craft a practicable and innovative concept to support appropriate HH in the NICU. Without having to discern complex engineering or technical competencies, this project’s nudging-based intervention is both simple and user-friendly. This study provides justification for continued investigation on which HH moments should be prioritized based on the clinical setting and how innovative, user-friendly improvement strategies can be designed to optimize HH compliance. A future randomized study is currently being planned to evaluate the effectiveness of our concept on compliance with HH and incidence of hospital-acquired infections in clinical practice.

## Supplementary Information

Supplementary Material 1

## Data Availability

The datasets generated during and/or analyzed during the current study are available from the corresponding author upon reasonable request.

## References

[1] Pittet D (2000). Effectiveness of a hospital-wide programme to improve compliance with hand hygiene. Lancet.

[2] Pittet D (2004). Hand hygiene among physicians: performance, beliefs and perceptions. Ann. Intern. Med..

[3] Pittet D (2001). Improving adherence to hand hygiene practice: a multidisciplinary approach. Emerg. Infect. Dis..

[4] Helder OK (2010). The impact of an education program on hand hygiene compliance and nosocomial infection incidence in an urban neonatal intensive care unit: an intervention study with before and after comparison. Int. J. Nurs. Stud..

[5] Erasmus V (2010). Systematic review of studies on compliance with hand hygiene guidelines in hospital care. Infect. Control. Hosp. Epidemiol..

[6] Marra AR, Edmond MB (2014). New technologies to monitor healthcare worker hand hygiene. Clin. Microbiol. Infect..

[7] Ward MA (2014). Automated and electronically assisted hand hygiene monitoring systems: a systematic review. Am. J. Infect. Control..

[8] Mukerji A (2013). An observational study of the hand hygiene initiative: a comparison of preintervention and postintervention outcomes. BMJ Open..

[9] Ivers N (2012). Audit and feedback: effects on professional practice and healthcare outcomes. Cochrane Database Syst. Rev..

[10] Thaler, R. H. & Sunstein, C. R. *Nudge: Improving Decisions about Health, Wealth, and Happiness* (Yale University Press, 2008).

[11] van Roekel H, Reinhard J, Grimmelikhuijsen S (2020). Improving hand hygiene in hospitals: comparing the effect of a nudge and a boost on protocol compliance. Behav. Public Policy.

[12] Nagtegaal R, Tummers L, Noordegraaf M, Bekkers V (2019). Nudging healthcare professionals towards evidence-based medicine: a systematic scoping review. J. Behav. Public Administration.

[13] Jansen SJ (2022). A longitudinal analysis of nosocomial bloodstream infections among preterm neonates. Eur. J. Microbiol. Infect. Dis..

[14] Jenner EA, Watson PWB, Miller L, Jones F, Scott GM (2002). Explaining hand hygiene practice: an extended application of the Theory of Planned Behavior. Psychol. Health Med..

[15] Whitby M, McLaws M, Ross MW (2006). Why healthcare workers don’t wash their hands: a behavioral explanation. Infect. Control. Hosp. Epidemiol..

[16] Ajzen I (1991). The theory of planned behavior. Organ. Behav. Hum. Decis. Process..

[17] Montano DE, Kasprzyk D (2015). Theory of reasoned action, theory of planned behavior, and the integrated behavioral model. Health Behav. Theory, Res. Pract..

[18] Glanz, K., Rimer, B. K. & Viswanath, K. *Health behavior: Theory, Research, and Practice* (John Wiley & Sons, 2015).

[19] Glanz K, Bischop DB (2010). The role of behavioral science theory in development and implementation of public health interventions. Annu. Rev. Public. Health.

[20] Karsh B, Holden RJ, Alper SJ, Or CKL (2006). A human factors engineering paradigma for patient safety: designing to support the performance of the healthcare professional. Qual. Saf. Health Care.

[21] Institute of Medicine (US) Committee on Quality of Health Care in America, Corrigan, J.M., Kohn, L.T. *To Err is Human: Building a Safer Health System* (National Academy Press (US), 2000). 25077248

[22] Chen W, Tseng CL (2021). What are healthcare workers’ preferences for hand hygiene interventions? A discrete choice experiment. BMJ Open..

[23] Gould D (2022). The problem with ‘My five moments for hand hygiene’. BMJ Qual. Saf..

[24] Sax H (2007). My five moments for hand hygiene: a user-centred design approach to understand, train monitor and report hand hygiene. J. Hosp. Infect..

[25] Yin S, Lim PK, Chan YH (2019). Improving hand hygiene compliance with patient zone demarcation: more than just lines on the floor. J. Patient Saf. Risk..

[26] Levchenko AI, Boscart VM, Fernie GR (2011). The feasibility of an automated monitoring system to improve nurses’ hand hygiene. Int. J. Med. Inform..

[27] Swoboda SM, Earsing K, Strauss K, Lane S, Lipsett PA (2004). Electronic monitoring and voice prompts improve hand hygiene and decrease nosocomial infections in an intermediate care unit. Crit. Care. Med..

[28] World Health Organization. *WHO Guidelines on Hand Hygiene in Health Care, First Global Patient Safety Challenge: Clean Care is Safer Care* (World Health Organization, 2009). 23805438

