# Supplementary material for: Developing a design-based concept to improve hand hygiene in the neonatal intensive care unit
Source: Pediatr Res. 2023 Jan 24;94(2):450–7. doi: 10.1038/s41390-023-02482-9 (PMC10382316; doi:10.1038/s41390-023-02482-9)
Supplement: Supplementary file 1 — Supplementary Material 1 [file 41390_2023_2482_MOESM1_ESM.pdf]

Supplementary Material 1. Procedural Aspects of Focus Group Sessions

Preparation sessions prior to each individual focus group session were held during which the following procedural components were covered: (i) the training of basic moderator skills including techniques for involving participants, keeping the discussion on track and ensuring sufficient depth and coverage, (ii) the development of a question guide, (iii) and the determination of strategies for analyzing the data. By exploiting both moderators' backgrounds, the collection of rich qualitative data was thereby maximized.

During the focus groups, three visualizations of proposed interventions aimed at improving HH compliance were used as a collective starting point to encourage open-ended discussions (Supplementary Material 2A-C). Participants were asked to express their opinions about the interventions and brainstorm about possible adjustments that could help increase the usability of the proposed interventions. Ideation was encouraged by design methods including brainwriting and braindrawing,[1] rapid prototype construction, demonstration of the proposed interventions and interactive task performance in which participants were asked to indicate preferred placements of a nudge on an incubator with color-adhesive notes. All remarks and insights gathered during the sessions were documented, with the collected data subsequently organized, analyzed and interpreted by the focus group moderators (SJ and BM). No software was used for the analysis of the qualitative data.

Supplementary Material 2A-C. Visualization of Proposed Interventions used during Focus Group Sessions

A.

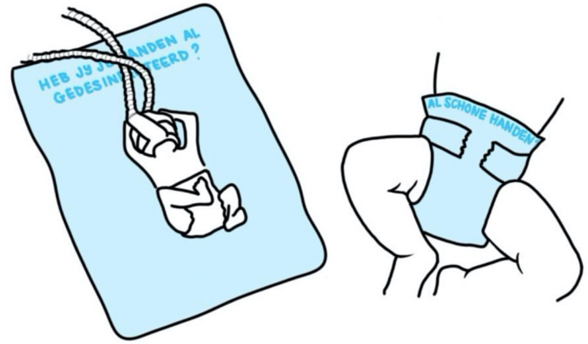

C.

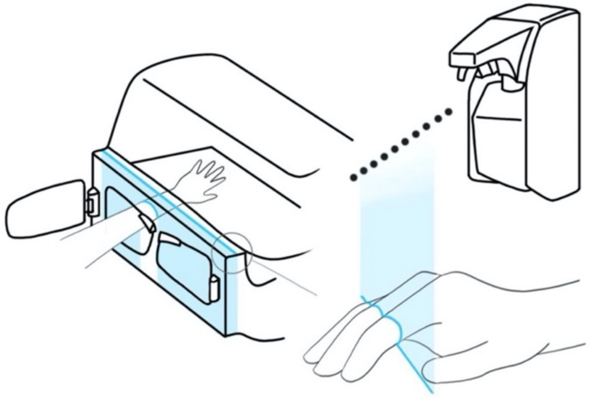

B.

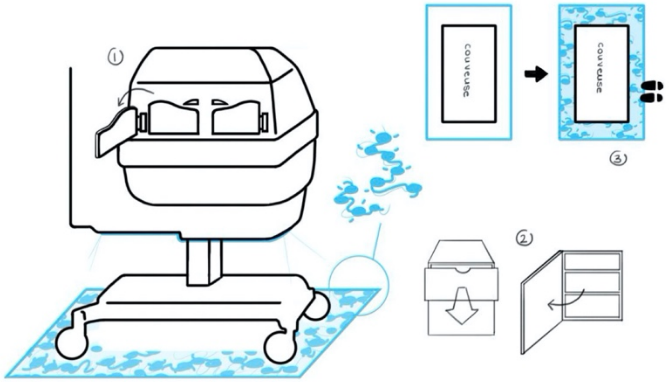

References

1. van Boeijen, A., Daalhuizen, J., Zijlstra, J. *Delft design guide: Perspectives, models, approaches, methods*. (BIS Publishers, 2020).
